# Recent Progress on Electromagnetic Field Measurement Based on Optical Sensors

**DOI:** 10.3390/s19132860

**Published:** 2019-06-27

**Authors:** Jun Peng, Shuhai Jia, Jiaming Bian, Shuo Zhang, Jianben Liu, Xing Zhou

**Affiliations:** 1 School of Mechanical Engineering, Xi’an Jiaotong University, Xi’an 710049, China; 2 State Key Laboratory of Power Grid Environmental Protection, China Electric Power Research Institute, Wuhan 430074, China

**Keywords:** electromagnetic field measurement, optical sensors, Faraday effect, magnetostriction, magnetic fluid, electro-optic effect, piezoelectric effect, electrostatic attraction

## Abstract

Electromagnetic field sensors are widely used in various areas. In recent years, great progress has been made in the optical sensing technique for electromagnetic field measurement, and varieties of corresponding sensors have been proposed. Types of magnetic field optical sensors were presented, including probes-based Faraday effect, magnetostrictive materials, and magnetic fluid. The sensing system-based Faraday effect is complex, and the sensors are mostly used in intensive magnetic field measurement. Magnetic field optical sensors based on magnetic fluid have high sensitivity compared to that based on magnetostrictive materials. Three types of electric field optical sensors are presented, including the sensor probes based on electric-optic crystal, piezoelectric materials, and electrostatic attraction. The majority of sensors are developed using the sensing scheme of combining the LiNbO_3_ crystal and optical fiber interferometer due to the good electro-optic properties of the crystal. The piezoelectric materials-based electric field sensors have simple structure and easy fabrication, but it is not suitable for weak electric field measurement. The sensing principle based on electrostatic attraction is less commonly-used sensing methods. This review aims at presenting the advances in optical sensing technology for electromagnetic field measurement, analyzing the principles of different types of sensors and discussing each advantage and disadvantage, as well as the future outlook on the performance improvement of sensors.

## 1. Introduction

Electromagnetic field (EMF) sensors have been drawing attention in recent years because of these wide usages in various areas, including navigation and medical instruments, and the power industry. For example, in magnetic resonance imaging diagnostics, magnetic field sensors are used for magnetic resonance data acquisition and assessment of magnetic radiation risk to protect workers from heavy occupational exposure [1,2,3]. Electromagnetic sensors are an effective tool to diagnose the troubles and ensure electronic equipment safety and stable operation in the power grid [4,5].

One type of conventional magnetic field sensors, a common tesla meter, consists of a hall sensor probe and a dashboard, which is not sensitive enough to measure the weak magnetic field (<1 mT) [6,7]. Another type of sensors consists of a coil with hundreds of fine-gage wires. When placed in an alternating magnetic field, an induced current is proportional to the strength of the applied magnetic field, which is not applicable to a static magnetic field. A commonly-used method for electric field measurement is based on a displacement current sensor which consists of two thinly-conductive plates connected together electrically. Another type of electric sensor is composed of a shield sector sheet and a fixed induction metal sheet. In the presence of an electric field, the shield sector sheet rotating the fixed metal sheet generates an induced current. The induced current is measured to derive electric field strength. The above methods have to use the metal antennas or probes to induce the electric field [8,9]. The metallic structures are susceptible to electromagnetic noise and can only measure EMF at a single point. 

Optical sensors have a number of advantages over conventional sensing techniques, such as compact size, strong adaptability to harsh environments, and immunity to electromagnetic interference, so lots of work has focused on EMF optical sensors in the decades at the end of last century [10,11,12]. One type of optical sensors are bulk sensors, which are integrated by a fiber with the field transducers, such as magneto-optic crystal, magnetostrictive materials, magnetic fluid, electro-optic crystals, and piezoelectric ceramics. The other type of sensor is an all-fiber sensor, where the fiber is not only used for light guiding but the sensing element. The all-fiber sensors for magnetic field measurement are based on the Faraday effect of magneto-optic fiber. The electric field all-fiber sensors are based on the electrostrictive effect of silica fiber, which is rarely used due to the low-sensitivity to electric field. Over the past decades, as the advances of new functional materials, such as magneto optic fiber, giant magnetostrictive materials, and magnetic fluid, the optical sensing technology for EMF has made great progress. The optical sensing technology also has a rapid development, especially the optical fiber sensing technique. There is a report about the optic fiber magnetic field sensor based on MF [13], but to our knowledge, no reviews specifically pay attention to the development of EMF optical sensors in the past ten years. Therefore, this paper aims to provide a summarized overview of the various types of EMF optical sensors. 

## 2. Magnetic Field Optical Sensors 

### 2.1. Probes Based on the Faraday Effect

The Faraday effect is a magneto-optical phenomenon—that is, an interaction between light and a magnetic field in a medium. The Faraday effect causes a rotation for the plane of polarization when plane-polarized light passes through a substance placed in a magnetic field. For a given substance, the rotation angle is proportional to the length traversed by the light and the magnetic field strength, which can be expressed as in [14]:

(1) $$\theta =V\int_{0}^{l}B(l)dl$$

where, ***θ*** is the rotation angle, ***V*** is the Verdet constant of a given substance or a function of concentration, temperature, and frequency, ***B*** is the magnetic flux density (***B*** = *μ*·***H***, ***H*** the magnetic field strength), and *l* is the length over which the magnetic field and light interact.

**Figure 1:**
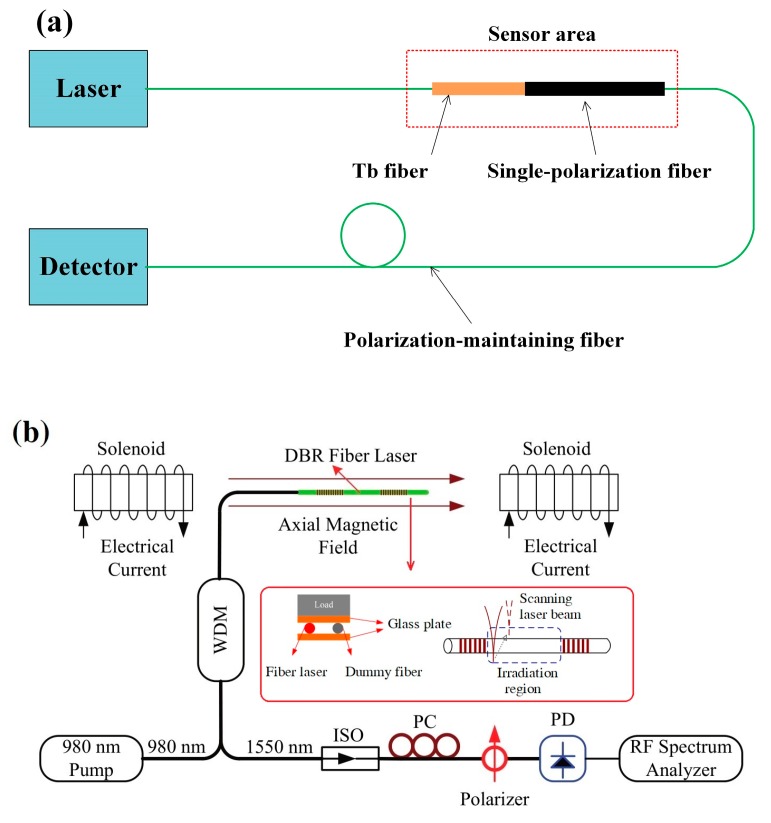
(**a**) Sensing system of a magneto-optic fiber magnetic-field sensor [15] (Copyright © 2010 OSA, Reprinted with permission) and (**b**) sensing system based on the heterodyning fiber grating laser [19] (Copyright © 2013 OSA, Reprinted with permission).

Sun et al. proposed an all-fiber optical magnetic-field sensor based on the Faraday rotation in terbium-doped (Tb) fiber. The Verdet constant of the Tb fiber is about 20 times larger than that of silica fiber, so this sensor probe has a higher sensitivity. Linear-polarized light is transmitted to the Tb fiber through polarization-maintaining fiber (PMF). The polarization of light rotates when the Tb fiber is placed in a magnetic field. The polarization rotation angle is related to the magnetic field strength, so the magnetic field can be measured by monitoring the output light intensity. The probe configuration and sensing system are shown in Figure 1a [15]. Some magnetic field sensors were designed using a heterodyning fiber grating laser. When a magnetic field is applied to the fiber grating laser, the birefringence in the fiber grating laser cavity becomes an elliptical birefringence. The Faraday rotation effect induces the circular birefringence into the cavity. The beat frequency of signal generated by two orthogonally polarized laser outputs is related to the magnetic field intensity [16,17,18]. In order to enhance the sensitivity of these magnetic sensors, Cheng et al. achieved the sensitivity enhancement by transverse force action or CO_2_-laser treatment to change the linear birefringence, as illustrated in Figure 1b [19]. Han et al. positioned a silicon steel sheet close to the fiber grating laser to strengthen the magnetic field intensity around the fiber grating laser cavity, wherein the silicon steel sheet worked as a magnetic field concentrator [20].

**Figure 2:**
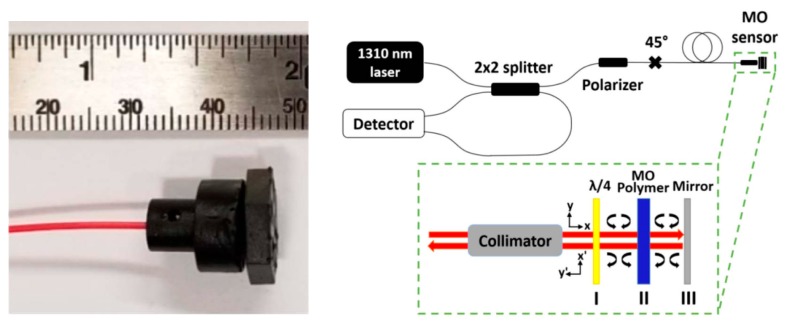
Magnetic field sensor prototype based on nanocomposite polymers and the sensing system [22] (Copyright © 2018 OSA, Reprinted with permission).

Descamps et al. proposed a magnetic field sensing method through detection of the circular birefringence of optical fiber induced by the Faraday effect. In an applied magnetic field, the FBG polarization properties, such as polarization dependent loss (PDL) and differential group delay (DGD), are changed. The magnetic field strength can be obtained by computing the diattenuation vector. It is a new demodulation technique allowing the use of a FBG written in common single-mode fiber (SMF) [21]. Amirsolaimani et al. developed a high sensitivity magnetometer using nanocomposite polymers, as shown in Figure 2. The Faraday rotation inside the nanocomposite generates the phase shift between two orthogonal polarization lights. The nanocomposite polymers were fabricated by encapsulating the Dy^3+^-doped magnetite and cobalt ferrite nanoparticles within polymethylmethacrylate matrix. The nanocomposite has a high Verdet constants (6.6 × 10^6^ °/T·m), which is about 10^5^ times larger than that of common silica fiber. Additionally, the design of a double optical path through the magneto-optic polymer enhances the Faraday rotation. In the contribution of the magneto-optic polymer with high Verdet constants and the double optical path design, the sensor can achieve a high sensitivity of 20 fT/√Hz. This sensing scheme can be used for detecting neuronal activity inside the brain [22].

Some distributed magnetic field sensors were presented based on the Faraday rotation. The magnetic field can be obtained by analyzing the state of polarization of the Rayleigh backscattered light. The polarization-sensitive optical frequency domain reflectometer (OFDR) is adopted to measure the state of polarization. Due to the faintness of the Faraday rotation in a common optical fiber, the sensors are most suited for intense magnetic fields, such as the application of magnetic resonance imaging [23,24].

### 2.2. Probes Based on Magnetostriction

Magnetostriction is a unique property of ferromagnetic substance. The energy of a ferromagnetic substance comprises three parts, the exchange, anisotropy and magnetostatic energies. The three magnetic energies variation induce the variation in volume, linear length, and shape, respectively. When a ferromagnet is magnetized in a magnetic field, a deformation will be generated [25]. At the end of last century, there was a great advance in ferromagnetic materials. Giant magnetostrictive materials (GMM) were discovered, such as Terfenol-D and Galfenol, which have larger deformation in a given magnetic field [26,27,28]. Lots of sensors exploit GMM as a magnetic field transducer. The fiber Bragg gratings (FBG) are attached to GMM, and then the stress induced by the magnetic field applies on the FBG. The magnetic field can be measured by detecting the Bragg wavelength shifts. 

**Figure 3:**
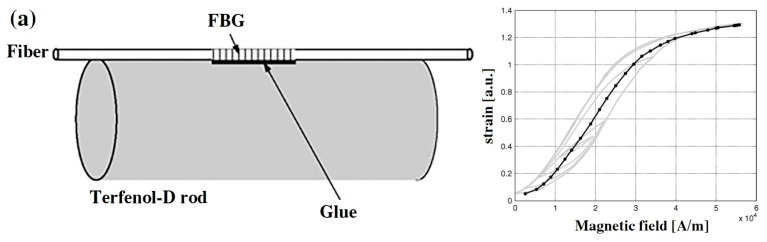

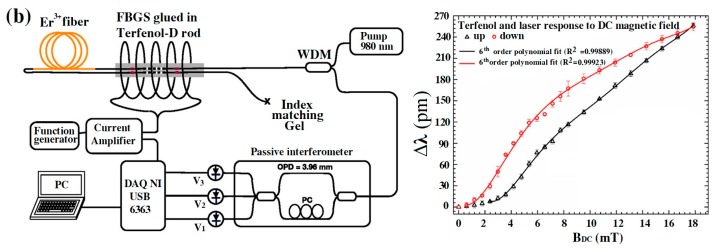
(**a**) Magnetic field sensor probe based on fiber Bragg gratings (FBG) and Terfenol-D rod and the performance of sensor [29] and (**b**) sensor sensing system based on FBG laser and Terfenol-D rod the test results [30]; permission conveyed through the Copyright Clearance Center, Inc.

The first type of sensors are designed by using GMM rods as the magnetic field transducer, and FBGs are bonded to the rod, which consist of a magnetic field probe. In [29], a magnetic field sensor was designed using a GMM rod and a FBG. The FBG is bonded to a Terfenol-D rod, as a sensor probe (Figure 3a). The magnetic field deforms the Terfenol-D rod, which stretches FBG to generate strain. The magnetic field can be detected by demodulating the wavelength of FBG. They also designed a compensation algorithm to achieve a linear response of this sensor. Nascimento et al. proposed a magnetic field sensor using an erbium-doped fiber optic laser and a Terfenol-D rod. A pair of FBGs are glued on the Terfenol-D rod to tune the laser wavelength (Figure 3b). The change in magnetic field can cause a laser wavelength shift. In their results, the wavelength shift is nonlinear to magnetic field intensity [30]. 

**Figure 4:**
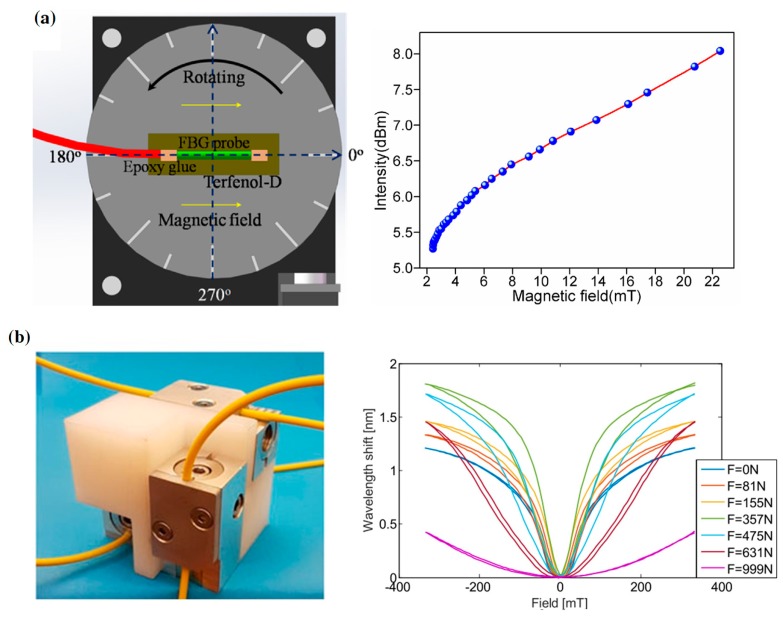
(**a**) Sensor head based on FBG glued on the Terfenol-D bar and the performance of sensor [31] and (**b**) tri-axial sensor and the wavelength shift versus magnetic field curves with different preloads [32]; permission conveyed through the Copyright Clearance Center, Inc.

A compact fiber magnetic field sensor was developed based on a phase-shifted fiber Bragg grating (PS-FBG) and a Terfenol-D bar. The PS-FBG is bonded to the Terfenol-D bar by epoxy resin as the sensing probe (Figure 4a). In the presence of a magnetic field, deformation of the Terfenol-D bar induces the Bragg wavelength shift of PS-FBG. The magnetic field strength can be measured by detecting the intensity variations of the reflected spectrum [31]. Filograno et al. designed a three-axis fiber optic magnetic field sensor for magnetic resonance imaging applications (Figure 4b). The uniaxial element of sensor is a FBG fixed on a block of Terfenol-D. The tri-axial sensor consists of three mutually perpendicular uniaxial elements. They tested the performance of the sensor and studied the effect of prestress on the wavelength shift. The results demonstrate that the slope of the wavelength shift is maximum in the range from −100 mT to 100 mT when the preload is 375 N [32]. 

**Figure 5:**
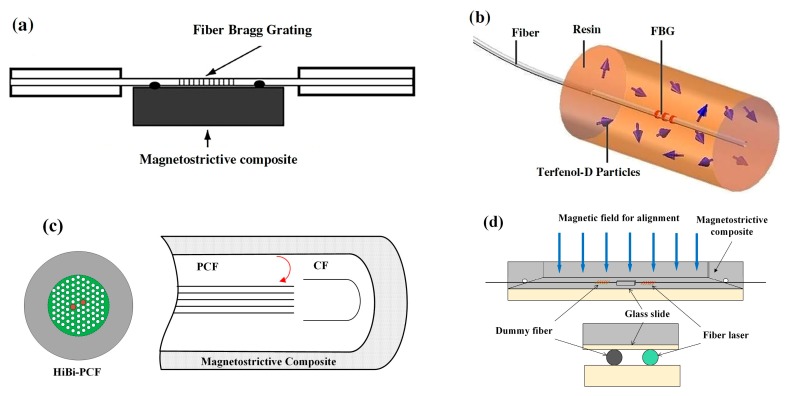
(**a**) Sensor head based on FBG bonded to magnetostritive composite [33], (**b**) senor probe consisting of FBG coated by magnetostritive composite [34], (**c**) sensing area photonic crystal fiber (PCF) coated by magnetostrictive composite [35], (**d**) configuration of sensor area based on the fiber-gratings laser embedded to the composite [36].

The second type of magnetic field sensors are integrated with magnetostrictive composite and FBGs. The magnetostrictive composite consists of Terfenol-D particles and an epoxy resin matrix. Liu et al. proposed a magnetic field sensor using magnetostrictive composite and FBG. The FBG is bonded to the surface of the composite, as illustrated in Figure 5a. Their results demonstrate that the peak wavelength of the sensor increases appropriately linearly with the increase of the magnetic field strength [33]. Quintero et al. presented a sensor composed of FBG and magnetostrictive composite. The FBG is coated by a thick layer of a magnetostrictive composite (Figure 5b). Terfenol-D particles are dispersed in an epoxy resin matrix. They tested the effect of volume fraction of Terfenol-D particles and particle size on sensor performance. The results show that the sensor has a resolution of 0.3 mT when the volume fraction of Terfenol-D particles (212~300 µm) is 30% [34]. They also designed a magnetic field sensor using photonic crystal fiber (PCF) coated by magnetostrictive composite (Figure 5c). The optical phase variation is a function of the change in magnetic field strength. The sensor can measure the magnetic field through detecting the wavelength shift [35]. He et al. embedded a heterodyning fiber-grating laser into magnetostrictive composite (Figure 5d). When a magnetic field is applied, the deformation stress of magnetostrictive composite acts on the heterodyning fiber laser to change the beat frequency. The response of this sensor shows a good directivity, a sensitivity of 10.5 Hz/μT, and a large measurable range up to about 0.3 T [36,37].

**Figure 6:**
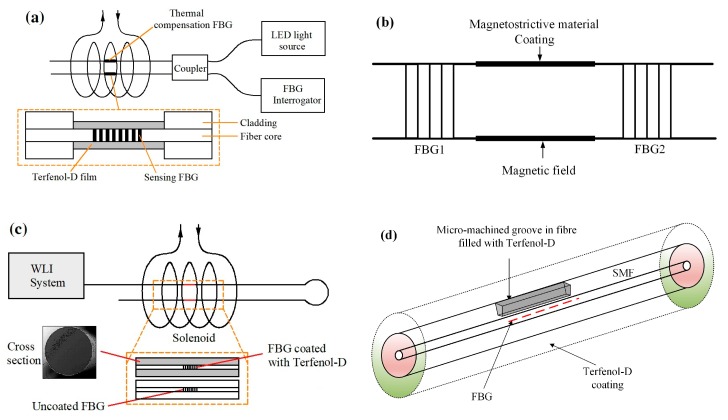
(**a**) Sensing head and system based on etched FBG coated by Terfenol-D film [38], (**b**) sensor probe composed of FBG Fabry–Perot cavity [39], (**c**) Thermal compensated magnetic-field sensor with coated and uncoated FBGs [40], (**d**) sensor head structure of FBG with micro-machined groove filed with Terfenol-D [42].

The third type of sensors were developed based on FBGs coated with a thin film of magnetostrictive materials. Yang et al. designed a sensor using corroded FBG coated with a layer of Terfenol-D (Figure 6a). The FBG was immersed in hydrofluoric acid solution to remove the cladding, and thin films of Terfenol-D were coated on the etched FBG through a sputtering system. They studied the effect of the etched diameter of FBG on the sensor sensitivity [38]. Chen and Li presented a magnetic field sensor based on FBG Fabry–Perot (FP) cavity ring-down technique. Two identical FBGs are written on a single mode fiber (SMF), as illustrated in Figure 6b. The section between two FBGs works as a FBG-FP cavity, which is coated by magnetostrictive material. In the presence of a magnetic field, the cavity is elongated by the magnetostriction, which causes the increase of cavity losses. The power transmission of the FBG-FP cavity is related to the magnetic field [39]. Silva et al. proposed a magnetic field sensor with thermal compensation. The sensing probe has a functional FBG coated with Terfenol-D and an uncoated FBG as a temperature compensation element, as shown in Figure 6c [40]. Schukar et al. developed a FBG sensor for magnetic field measurement. The FBG is coated by a magnetostrictive iron-nickel layer with an iron concentration of 50% [41]. A femtosecond laser-inscribed FBG sensor was proposed for detection of the magnetic field. A micro-machined groove is created in the fiber and then the grooved section is filled with Terfenol-D by RF sputter, as shown in Figure 6d [42]. 

Another type is distributed magnetic field sensors based on magnetostriction. Masoudi and Newson proposed a distributed optical fiber magnetic field sensor by measuring the strain on the fiber which was induced by a nickel wire bonded to the fiber. The sensing principle is based on the distributed dynamic strain measurement technique—optical time-domain reflectometry (OTDR). The fiber strain is induced by the magnetostriction of the nickel wire in the presence of a magnetic field. The phase difference between the backscattered light from the two sections of fiber is a function of the magnetic field. This sensor can detect multiple magnetic fields along a 1 km sensing fiber with a spatial resolution of 1 m [43]. Du et al. also presented a distributed magnetic field sensor based on the same principle. The magnetostrictive Fe–Co–V alloy film is attached to a 51 m-long SMF. The minimum detectable magnetic field variation is 12.9 mT, and the spatial resolution is 4 cm in the operating range from 12.9 mT to 143.3 Mt [44,45]. 

### 2.3. Probes Based on Refractive Index Tunability of Magnetic Fluid 

Magnetic fluid (MF), commonly called ferrofluid, is a stable colloidal solution of single domain magnetic nanoparticles. The MFs have a unique combination of normal liquid behavior and the ability to interact with the magnetic field. It has many remarkable properties, such as refractive index tenability, birefringence effect, dichroism effect, thermal effect, etc. [46]. The refractive index tenability of MF has been widely used in the design of optical sensors [47,48,49]. 

**Figure 7:**
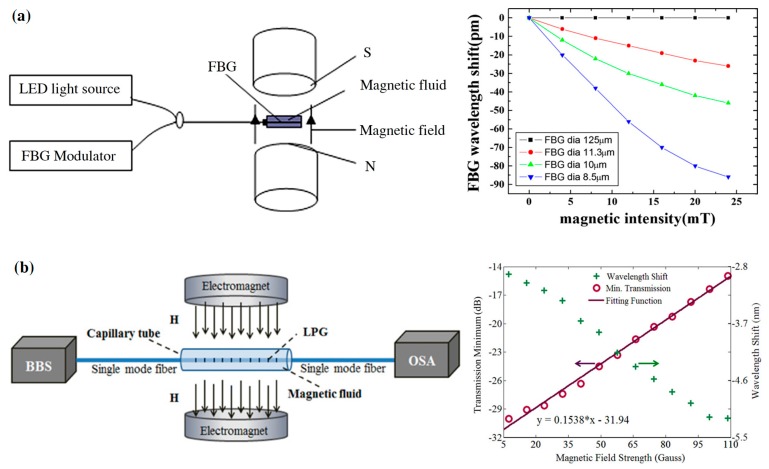
(**a**) Magnetic field sensing system based on etched FBG immersed into magnetic fluid (MF) and the responses of sensors with different FBG diameters to magnetic intensity [51] and (**b**) Sensing system based on Long period fiber grating (LFBG) and the relationship between output power of transmission light and magnetic field strength [52]; permission conveyed through the Copyright Clearance Center, Inc.

One type of optical fiber magnetic field sensors is proposed by using MF as the cladding of optical fiber structures. For example, the MF works as the cladding of FBG. Etched FBG is placed into a container filled with MF, and the sensing system is shown in Figure 7a. The effective refractive index of FBG is related to the refractive index of cladding (i.e., MF). The refractive index of inside MF is tuned by the external magnetic field. The magnetic field can be measured by detecting the reflected wavelength shifts. They tested the interaction between the etched FBG diameter and the sensitivity of the sensor. The results demonstrate that the sensor has a sensitivity of 2.3 pm/mT and 3.44 pm/mT [50,51]. Zhang et al. placed the Long period fiber grating (LFBG) into a cavity filled with MF as a magnetic field sensor probe, as illustrated in Figure 7b. The resonance wavelength of LFBG is related to the refractive indices of the core mode and the cladding mode, wherein the core mode does not change in a magnetic field. The magnetic field can be measured through detecting the power of the transmission spectrum [52]. Gao et al. used D-shape fiber with LPBG surrounded by MF as a sensing probe for magnetic field measurement [53]. Luo et al. proposed a magnetic field sensor using a hybrid LFBG encapsulated in a capillary tube filled in MF. The hybrid LFBG is fabricated by splicing the microstructure optical fiber to SMF [54]. Zheng et al. proposed a magnetic field sensor based on the interaction between tilted fiber Bragg grating (TFBG) and MF. The TFBG and MF are packaged in a capillary tube as a sensing probe. The transmission spectrum of TFBG correlates to the magnetic field strength [55]. Narasimman et al. demonstrated a magnetic field sensor combining the etched fiber and MF composed of Co-doped ZnO nanorods. They studied the effect of ZnO nanorods of different Co concentrations on the sensor sensitivity. The results show that the sensor has a maximum sensitivity of ~18% for Co-doped ZnO nanorods in the range from 17.2 mT to 180.8 mT [56].

**Figure 8:**
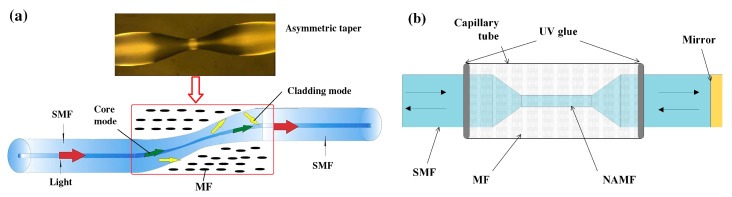
(**a**) Schematic of sensing probe based on tapered fiber immersed into MF [57] and (**b**) sensor probe based on non-adiabatic tapered optical fiber [62]; permission conveyed through the Copyright Clearance Center, Inc.

Another type of optical magnetic field sensor is based on MF and an optical fiber interferometer. Deng et al. developed a fiber magnetic field sensor using MF and a tapered optical fiber structure (Figure 8a). This structure works as a core-cladding-mode interferometer (CCMI). The fiber taper and MF are encapsulated in a capillary tube as the sensing probe. When the probe is immersed in the magnetic field, the wavelength of the output spectrum will shift. Their results demonstrate that this sensor has good optical properties and a high sensitivity of 162.06 pm/mT in the range from 0 to 21.4 mT [57]. They also proposed a sensor based on MF and a tapered microstructured optical fiber (TMOF) structure. The structure is a section of micro-structured optical fiber with six holes filled with MF, and the two ends are spliced to SMFs. The refractive index variation of MF induced by the magnetic field contributes to effective refractive index differences between the core and cladding modes. The magnetic field can be measured by detecting the interference spectrum shift induced by refractive index variation. The sensor has a sensitivity of 117.9 pm/mT with a linear range from 0 mT to 30 mT [58]. The optical fiber magnetic field sensors were developed using the similar sensing principle. The probe is a section of tapered thin-core fiber encapsulated in capillary tube filled with MF [59,60]. There are reported sensors based on nonadiabatic tapered optical fiber (NATOF) and MF. The nonadiabatic tapered optical fiber has a high sensitivity to the refractive index, so the sensor is more sensitive to the magnetic field. The sensor proposed by Layeghi et al. has a sensitivity of −74.4 pm/mT, and a good linearity to magnetic field strength in the range from 0 to 44 mT [61]. Luo et al. adopted a non-adiabatically tapered microfiber (NATMF) to design a sensor, which has a high sensitivity of 1744 pm/mT, as shown in Figure 8b [62]. 

**Figure 9:**
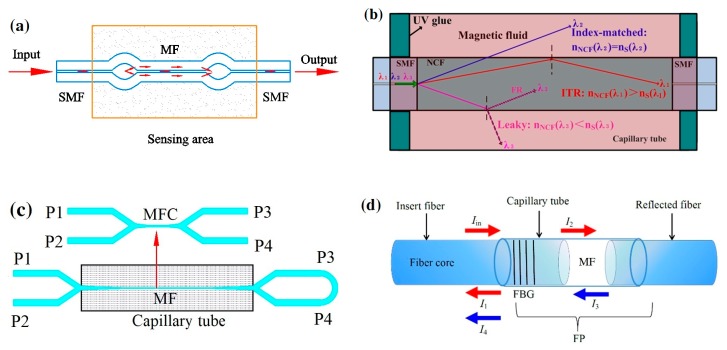
(**a**) Sensing arm of sensor based on fiber spherical-structure interferometer and MF [63], (**b**) sensor probe structure of single mode fiber (SMF)- no-core fiber (NCF)-SMF encapsulated in capillary tube filled with MF [67], (**c**) sensor probe based on the Sagnac loop with microfiber coupling structure [69], (**d**) sensing head based on F-P interferometer and MF [70].

There are magnetic field sensors proposed by combining fiber spherical structure interferometer and MF. The sensing arm consists of two special up-tapered joints on a SMF. MF is used as the cladding of the sensing arm (Figure 9a). The valley wavelength of the interference spectrum may shift as the refractive index changes. The magnetic field can be detected by monitoring the wavelength shift [63,64]. Tong et al. proposed a sensor based on another spherical structure fiber interferometer. The interferometer consists of down-taper and spherical structure, which is coated by MF identically [65]. Magnetic field sensors were developed based on a hybrid structure of SMF-no-core fiber-SMF (Figure 9b). A section of no-core fiber (NCF) is spliced between two pieces of SMFs and the structure is immersed in a capillary tube filled with MF. The coupling wavelength dip is related to the refractive index of MF dominantly. The external magnetic field generates the wavelength dip shift, and the magnetic field strength measurement can be achieved by observing the wavelength shift [66,67]. Jia et al. proposed a temperature self-compensative magnetic field sensor based on NCF cascaded with FBGs. The NCF is also sandwiched between two pieces of SMFs, which is coated with MF. The Bragg wavelengths of FBG do not change with the magnetic field, so the transmission intensity is determined by the SMF-NCF-SMF spectral shift. Due to the similar thermal effect of NCF and FBG, the cross-talk between temperature and the magnetic field is greatly reduced [68]. Pu et al. proposed a magnetic field sensor by combining the microfiber coupling structures with MF. They studied two different coupling configurations (Sagnac loop and knot resonator), and the sensing structure based on the Sagnac loop is shown in Figure 9c. The coupling area is encapsulated in a capillary tube, which is filed with MF. The sensor based on the knot resonator structure can achieve a high sensitivity of 1718 pm/mT [69]. Xia et al. adopted a FP-FBG structure to design a magnetic field sensor. Two SMFs are inserted into a capillary tube and a gap is kept between the two ends. A FBG is written on one end of SMF and the gap is filled with MF (Figure 9d). The sensor has a sensitivity of 530 pm/mT and the measurement resolution can reach 37.7 μT [70]. 

**Figure 10:**
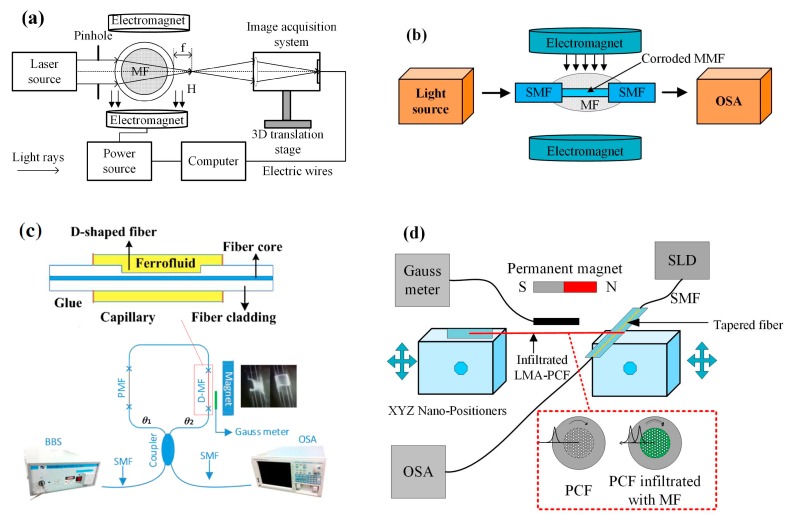
(**a**) Magnetic field sensing system based on a capillary tube filled with MF [71], (**b**) schematic of sensing system based on MF and a singlemode-multimode-singlemode (SMS) fiber structure [72], (**c**) sensor head and sensing system based on an optical fiber Sagnac interference effect [73], (**d**) sensing system configuration of photonic crystal fibers (PCF) infiltrated with MF [75]; permission conveyed through the Copyright Clearance Center, Inc.

Ji et al. proposed a magnetic field sensor, of which the probe is based on the capillary tube filled with MF. The capillary is similar to a cylindrical lens. When a magnetic field is applied, the focal line position of the cylindrical lens changes. The magnetic field can be measured through detecting the position of the focal line (Figure 10a) [71]. Wang developed a magnetic field sensor consisting of MF and a singlemode-multimode-singlemode (SMS) fiber structure. They spliced the SMFs to a two-ends of 12-mm-long multimode fiber, and then positioned the SMS structure into a HF acid to decrease the cladding diameter. The corroded SMS fiber structure is immersed into the MF, which works as a magnetic field sensor probe (Figure 10b). The sensor has a sensitivity of −168.6 pm/mT in the range from 12 mT to 32.5 mT [72]. Lei et al. reported a magnetic field sensor based on a D-shaped fiber and MF. The D-shaped slot is created in a common SMF, and then filled with MF. The demodulation system is based on a Sagnac interferometer (Figure 10c). The sensitivity of this sensor can reach 82.3 pm/mT in the range of 0.1–30.4 mT [73]. Jiang et al. designed a magnetic field sensor by using D-shaped fiber and MF. They deposited a gold film onto the side-polished region of the fiber to realize surface plasmon resonance. The sensitivity has a significant enhancement (up to 5987 pm/mT) due the high refractive-index sensitivity of the surface plasmon resonance (SPR) [74]. Mahmood et al. demonstrated a magnetic field sensor using a PCF infiltrated with MF. A piece of PCF is infiltrated with MF as a cylindrical whispering-gallery-mode micro-resonator. In the presence of a magnetic field, the whispering-gallery-mode resonances shift to a longer wavelength. The sensing system is shown in Figure 10d. The results illustrate that the sensor sensitivity is 110 pm/mT in the range from 0 to 38.7 mT [75]. 

A few distributed magnetic field sensors based on MF have been covered. There are a few studies on the distributed refractive index sensing [76]. Du et al. detected the refractive index of the external medium surrounding the fiber by analyzing the Rayleigh backscattering signals, wherein the OFDR technique is adopted. This concept can be used as a reference for the distributed measurement of the magnetic field based on MF.

## 3. Electric field Optical Sensors

### 3.1. Probes Based on the Electro-Optic Effect of Crystal

An electro-optic effect is a change in optical properties of a substance in response to an electric field. These property changes can be classified into two types: variation in absorption and refractive index. The application of electro-optic crystals in electric field measurement has drawn attention over recent years [77,78,79]. 

**Figure 11:**
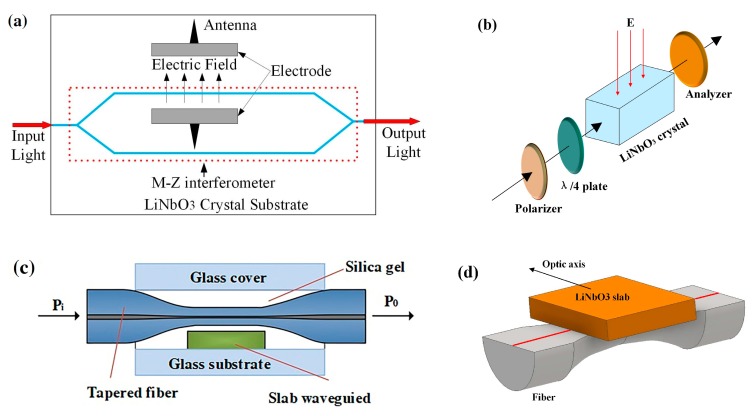
(**a**) Sensing area of electric field sensor based on Mach-Zehnder interferometer and LiNbO_3_ [80], (**b**) sensing unit based on Pockels effect of LiNbO_3_ [84], (**c**) sensor probe structure of tapered fiber and waveguide [85], (**d**) sensor head configuration of D-shaped fiber and LiNbO_3_ slab [89]; permission conveyed through the Copyright Clearance Center, Inc.

One type of optical electric field sensor is developed using electro-optic crystal as a waveguide to sense an electric field. The refractive index of crystal is changed in the presence of an electric field. Zeng et al. used LiNbO_3_ crystals to design an integrated electro-optic sensor for electric field measurement. The sensor area is a Mach-Zehnder interferometer (MZI), as shown in Figure 11a. Two parts of light interfere with each other by passing through the right coupling. Electrodes with an antenna are around one arm of the interferometer. When an electric field is applied to the antenna, a voltage is induced across the electrodes of the modulator. Then the change in refractive index of LiNbO_3_ crystal causes the change in phase of transmission light. The power of the sensor output signal is proportional to the applied electric field strength. They analyzed the performance of sensors with different electrodes. The results show that the mono-shield electrode sensor without an antenna is more efficient than those with antennas in measurable amplitudes [80,81,82]. Toney et al. designed an integrated optical electric field sensor by using a LiNbO_3_ crystal waveguide and a Mach–Zehnder interferometer. This sensor can achieve a minimum detectable field of 20 mV/m/√Hz at 100 MHz in the electric fields from 20 mV/m to 30 kV/m [83]. Yang et al. proposed an optical electric field sensor using a LiNbO_3_ crystal. The sensing unit is shown in Figure 11b. The phase shift of light transmitted in the crystal changes in an applied electric field. The sensor output is linear to the electric field strength in the range from −801 kV to 801 kV with a wide frequency band (10 Hz–10 MHz) [84]. 

Han et al. proposed an all-fiber electric field sensor based on tapered fiber and a LiNbO_3_ slab waveguide coupler (Figure 11c). The evanescent is coupled between a tapered fiber and a slab waveguide. The shift of the resonance wavelength is related to electric field intensity. The minimal resolution of this sensor is approximately 15 kV/m [85]. Seng et al demonstrated an electric field sensor based on a slab of LiNbO_3_ and D-shaped fiber. The D-fiber is etched to be a taper, and the slab is positioned closed to the tapered core, as shown in Figure 11d. Light couples between the D-fiber and the slab waveguide to generate a resonance wavelength. The change in the refractive index induced by the electric field causes the resonance wavelength shift. The electric field strength can be measured by detecting the wavelength shift [86,87,88,89]. Chadderdon et al. presented an electric field sensor using a coupling configuration between D-fiber and a waveguide slab. They chose an electro-optic polymer as the waveguide slab because the materials have the lowest RF permittivity and strong electro-optic effect. The sensor has a sensitivity of 0.147 pm/ kV [90]. 

**Figure 12:**
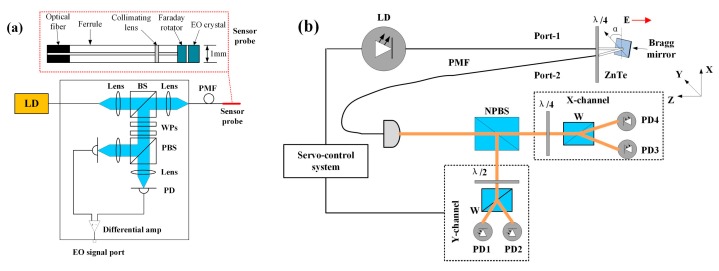
Sensor probe and sensing system (**a**) based on CdTe crystal [91] and (**b**) sensing system of two-dimensional electric field using ZnTe crystal [92].

Togo et al. designed an electric field sensor, which consists of an electro-optic crystal (CdTe), a Faraday rotator, a collimating lens, a ferrule, and a piece of PMF, as shown in Figure 12a. Polarized light is transmitted to the CdTe crystal through PMF and reflected by the dielectric mirror. When an electric field is applied to the CdTe crystal, birefringence is induced and the polarization of light changes. The polarization change is proportional to the electric field intensity. The performance tests show that the response of the sensor is stable with a fluctuation of less than 0.3 dB [91]. Gaeremynck et al. proposed an electro-optic sensor based on a ZnTe crystal, which can measure two orthogonal components of the electric field simultaneously (Figure 12b). The sensing area consists of a ZnTe crystal and a laser probe beam. An elliptically polarized light is split into two beams, and the polarization states of the two beams of light are characterized by the refractive index. In the presence of an electric field, the change in the refractive index of ZnTe crystal will induce the variation of phase [92]. Barbieri et al. used a Barium-Borate (BBO) crystal as a part of the electric field sensing unit. The birefringence of BBO crystal is proportional to the electric field strength. This sensor can measure two components of the electric field by a single anisotropic crystal [93]. 

**Figure 13:**
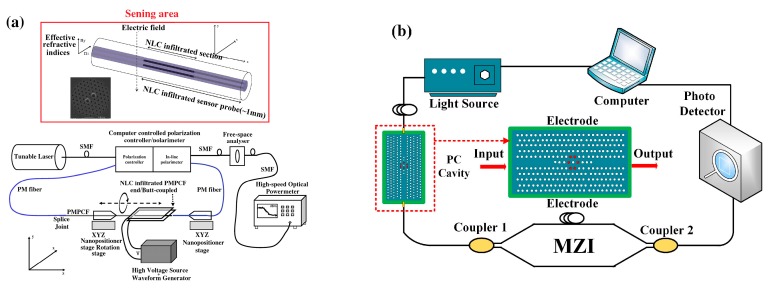
(**a**) electric field sensor probe and sensing system using polarization-maintaining photonic crystal fiber (PMPCF) and nematic liquid crystal (NLC) [94] and (**b**) sensing system based on photonic crystal cavity and liquid crystal (LC) [97]; permission conveyed through the Copyright Clearance Center, Inc.

The other type of electro-optic electric field sensor is proposed using the liquid crystal (LC) as the electric field sensing element. Mathews et al. developed an electric field sensor using a polarization-maintaining photonic crystal fiber (PMPCF) infiltrated nematic liquid crystal (NLC), as shown in Figure 13a. They studied the transmission properties for different lengths of the infiltrated section of PCF. The results demonstrate that the sensor has a sensitivity of 50 V/m in the range of 3.4–4.1 MV/m [94,95,96]. Zhao et al. developed an electric field sensor using a photonic crystal cavity infiltrated with LC (Figure 13b). An electric field applied can induce the resonance wavelength of a photonic crystal cavity shift. This wavelength shift is monitored by a MZI. Their results illustrate that the sensor can achieve a high sensitivity of 7 nW/(V/m) and a maximum resolution of 0.143 V/m [97]. 

**Figure 14:**
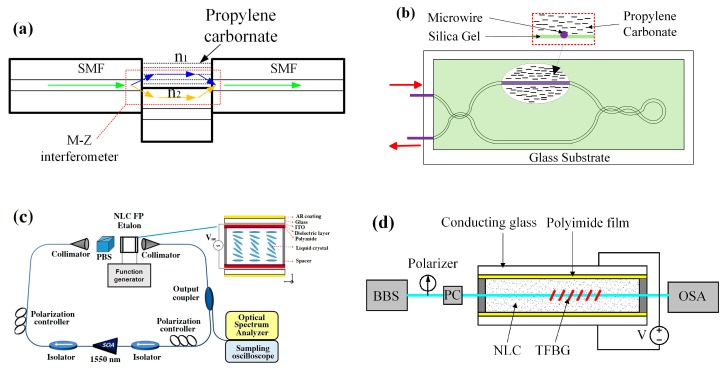
(**a**,**b**) Senor probe based MZI and propylene carbonate [98,99], (**c**) sensing area and system using he NLC FP etalon [100], (**d**) sensing system based on tilted fiber Bragg grating (TFBG) and NLC [104]; permission conveyed through the Copyright Clearance Center, Inc.

Zhu et al. designed a compact electric field sensor using an optical fiber interferometer. A short section of SMF is spliced between two sections of SMF with a large lateral offset of 62.5 μm, which can work as a MZI, as shown in Figure 14a. The open arm of the interferometer is filled with propylene carbonate. In the presence of an electric field, the refractive index of propylene carbonate will change, which causes interferometric fringe shifts. The electrical field can be acquired by monitoring the fringe shift [98]. Han et al. also presented an electric field sensor based on propylene carbonate and MZI (Figure 14b). The interferometer is composed of silica micro-wire. One arm of the interferometer is coated by propylene carbonate. The sensor can detect an electric field with a frequency of 50 Hz and an impulse electric field. The sensor can detect a minimum strength of 59 V/m for a 50 Hz electric field [99]. 

Fiber-optic electric field sensors were proposed using a NLC FP etalon-based multiwavelength-swept laser. The NLC FP etalon can be looked at as a wavelength filter by applying the electric field (Figure 14c). The transmission wavelengths change according to the electric field applied to the NLC FP etalon [100,101,102]. Tabassum et al. presented a sensitivity-enhanced electric field sensor based on the SPR. The sensing area is a section of corroded optical fiber immersed in a liquid dielectric medium, and the corroded region is coated by metal film. They analyzed the effect of coating metal materials on the performance of the sensor. The result illustrates that the sensor with bimetallic layers is better than the single metal layer ones [103]. Chen et al. proposed an electric field sensor based on TFBG immersed in liquid crystal. They embedded a TFBG into a NLC cell as a sensing probe (Figure 14d). In an electric field applied, NLC orientation change will cause the variation of its refractive index. The surrounding refractive index can be sensed by the TFBG. Their results demonstrate that the sensor can measure an electric field over a range of 100–480 kV/m with a sensitivity of 0.287 dB/kV/cm [104]. 

### 3.2. Probes Based on the Converse Piezoelectric Effect 

The piezoelectric effect is a unique property of materials where they will generate an electric field or current if subjected to physical stress. Commonly, piezoelectric materials include piezoelectric ceramic (PZT) and polyvinylidene fluoride (PVDF). The same effect can also be observed in reverse, where an imposed electric field on the crystal will put stress on its structure, which is the converse piezoelectric effect. The converse piezoelectric effect has been widely used for the design of electric field sensors. 

**Figure 15:**
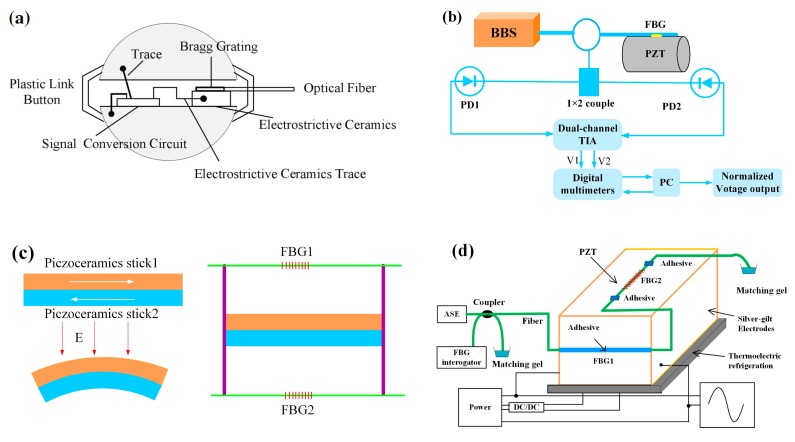
(**a**) Electric field sensor probe based on electrostrictive ceramics [106], (**b**) sensing system based on FBG bonded to a piezoelectric ceramic (PZT) cylinder [107], (**c**) sensing probe configuration of two FBGs and piezoceramics [108], (**d**) sensing system with high sensitivity using two FBGs glued on piezoceramics with different direction [109].

Marignetti et al. developed a FBG sensor to detect the electric field for the end windings of high-voltage electric machines. The working principle relies on the electrostrictive effect of silica. When an electric field is applied, a mechanical force generates a strain resulting in a wavelength shift of FBG. The field strength can be measured by demodulating the wavelength shift [105]. Zhao et al. presented an alternating current (AC) electric field sensor based on electrostrictive ceramics and FBG. The sensor head is composed of FBG attached to ceramics, two metal spherical shells, and a signal conversion circuit (Figure 15a). When an electric field acting on the metal spherical, the metal spherical shell induces an electric signal, and the signals are transformed and amplified. Then the transformed DC voltage signals apply on the ceramics and the stress induces a wavelength shift of FBG. Electric field strength can be measured through the detection of a wavelength shift [106]. Anirudh et al. proposed a FBG sensor for DC electric fields measurement. The electric field transducer is a PZT crystal, and the FBG is boned to PZT crystal. In presence of an electric field, PZT crystal deformation induces the wavelength shift of FBG. They also designed a wavelength interrogation system, as shown in Figure 15b [107].

Liu et al. demonstrated a FBG electric field sensor with two of the same piezoceramics blocks glued together with opposite directions. Two rods are fixed on the two ends of piezoceramics blocks respectively, and two FBGs are glued to the rods (Figure 15c). When an electric field is applied to the piezoceramics, the two piezoceramics blocks will deform inversely and bend to the same side, which induces the wavelength shift of two FBGs [108]. Yao et al. developed an electric field sensor based on two FBGs adhered to a piezoelectric ceramic block (Figure 15d). FBG1 is glued on the surface of the piezoceramics along the d_33_ polarized direction, and FBG2 is glued along the d_31_ polarized direction. The difference of the Bragg wavelength of FBG1 and FBG2 is regarded as the output, which can enhance the sensor sensitivity and reduce the cross-talk of temperature [109]. Floridia et al. used a similar method to propose a temperature-independent FBG sensor for electrical field measurement. The sensing probe is a FBG bonded to piezoelectric substrate, and another FBG is attached to an identical piezoelectric substrate as temperature compensation [110]. 

### 3.3. Probes Based on Electrostatic Attraction 

**Figure 16:**
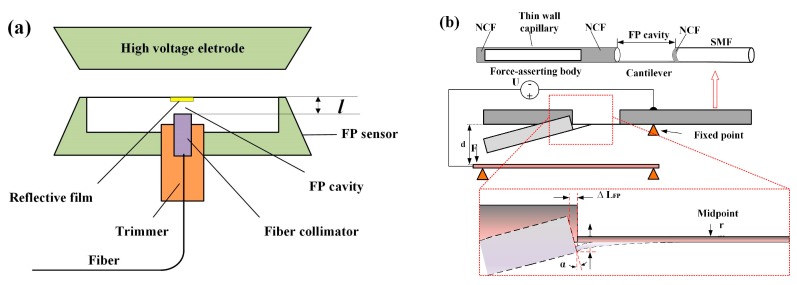
(**a**) Schematic of sensor probe [111], (**b**) configuration of sensor head and the operating principle [112].

A conductor is placed in an electric field, the free charge carriers inside the conductor redistribute, in contrast with the lattice-bound opposite charges. Two surfaces with opposite charges will generate an attractive electric force. Zhang et al. designed a high voltage electrostatic sensor based on the Fabry–Perot interferometer (FPI) (Figure 16a). The FP cavity of the sensor is formed by an optical collimator and polyester film aluminized outside. When a high voltage is applied on the electrode, an electric field will be generated. The polyester film is deformed by an electrostatic force, which causes the change in length of the F-P cavity and the output spectrum shift of the sensor [111]. Javernik and Donlagic proposed a fiber-optic voltage sensor based on the principle of electric charge attraction. The sensor head consists of a SMF fiber, a section of NCF, a micro-cantilever, and a force-asserting body, as illustrated in Figure 16b. The force-asserting body is coated by conductive transparent material. When the sensor is connected to the voltage/potential, an attractive electric force will deflect the micro-cantilever. The deflection causes the change in length of the F-P cavity and induces the F-P peak wavelength shift. They claimed that this sensor could be used for DC and AC power-grid frequency voltage measurements [112]. 

**Figure 17:**
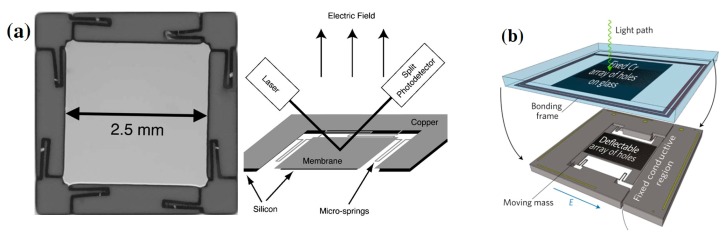

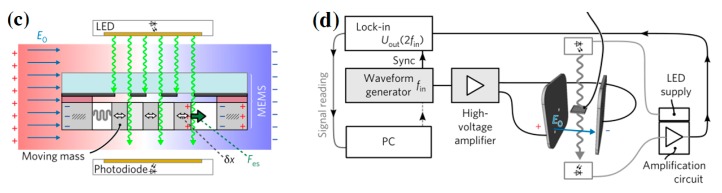
(**a**) Photograph of micro-spring supported copper membrane and the schematic of operating principle [113], (**b**–**d**) MEMS chip, working mechanism and the sensing system [114]; permission conveyed through the Copyright Clearance Center, Inc.

Roncin et al. developed an electric field sensor using a micro-spring supported copper membrane (Figure 17a). The sensing principle is based on the electrostatic force to deflect the copper membrane and an optical sensor to detect the membrane movement. Their result shows that the sensor can detect a minimum AC field of 0.3 V/m at 97 Hz when a 17 kV/m DC bias field is applied [113]. Andreas Kainz et al. developed an optical microelectromechanical system (MEMS) sensor for electric field measurement. The sensor structure is a silicon microstructure, as shown in Figure 17b. The sensor configuration is composed of a fixed layer and a movable layer. The fixed layer is a glass plate with Cr film, which works as an optical shutter. The movable layer is a spring-suspended Si plate, and the moving mass part could be displaced by the electrostatic force when an electric field is applied. A beam of light passes through the fixed layer and the moving layer successively, and then is detected by a photodiode. The displacement of the moving mass part can be read out by detecting the light flux. The electric field strength is related to the output of photodiode. The sensor has an electric field resolution of 100 V/m/√Hz with a measurement range of tens of kilovolts per meter [114]. 

## 4. Conclusions and Outlook

In this review, a variety of sensing configurations of electromagnetic field sensors were demonstrated. The performance parameters of these sensors are shown in Table A1 and Table A2 in the Appendix A. Magnetic field sensors were summarized into three types according to the sensing methodology of sensor probes. Wherein, the probes based on the Faraday effect originates earliest, and have a complex sensing system, including lots of optical elements, such as the Faraday rotator, polarizer, PMF, etc. The sensor sensitivity is determined by the Verdet constant. The Verdet constant of common silica fiber is very small, which makes it not suitable for weak magnetic field strength. The detection variable is the rotation angle of polarization light, it has strong resistance to interference, and can work in unshielded environments. Because the Verdet constant is almost the only limitation to the sensitivity, the sensitivity can be enhanced easily through selecting the magneto-optic materials with large Verdet constant. Therefore, it can achieve a large detecting range for magnetic field.

The sensing method based on magnetostriction developed with the rising of giant magnetostrictive materials. The sensor element has merits of easy fabrication, compact size and strong environment adaptability. The magneto-elastic materials have intrinsic hysteresis phenomena, meaning these sensors cannot monitor the field with high frequency. The deformation of GMM is relatively small under a small applied magnetic field, and the deformation has a definite linear range. This sensing scheme is suitable for the measurement of stationary and large magnetic fields (<500 mT). 

The sensing principle based on refractive index tunability of magnetic fluids has become a hotspot for magnetic field measurement in recent years, because of its high sensitivity, compact size, and flexibility in combination with various optical fiber structures, such as etched fiber gratings (FBG, LFBG, TFBG), fiber taper, F-P cavity, and other fiber structures. However, the detecting range is limited by the tunable range of the refractive index of magnetic fluid. The operating range of this type of sensor is relatively small (<100 mT). There is cross-talk between temperature and the magnetic field, so the thermal compensation should be taken into consideration in real applications. 

The electric field sensors are classified into three types according to the sensing principles. The principle, based on the electric-optic effect of crystal, is the mainstream sensing scheme due to the good electro-optic properties of the crystal. These sensors usually have integrated probes, which is a combination of electro-optic crystals with fiber interferometric structures. The interferometric structure-based probe is highly sensitive to the electric field, but susceptible to external interference. The sensing method based on piezoelectric ceramics has a simple structure and easy fabrication, but it has low sensitivity due to small deformation in the presence of a weak electric field. The sensing principle based on electrostatic attraction is a less commonly-used sensing method, but this is a promising alternative to optical MEMS sensors for electric field measurement. 

Most of the above-discussed sensors are single point measurement, seldom involving distributed measurement. Further work can focus on the distributed measurement because of the intrinsic advantages of optic fiber sensing technology. Fully distributed electric or magnetic field measurement could have a huge impact on many applications, such as the power industry. Currently, there are no reported optical sensors on the simultaneous sensing of electric and magnetic fields. In the future, the optical sensors for simultaneous measurement of electromagnetic fields can be taken in consideration, because both fields exist simultaneously sometimes. 

## Appendix A

Aimed to give a clear performance comparison, this appendix demonstrates a table of the main sensing parameters of the above-discussed sensors.

**Table A1:** Performance parameters of optical fiber magnetic field sensors.

Detection Mechanism	Sensor Probe Configuration	Field Transducer	Probe Size/mm	Detecting Range	Sensitivity/Resolution	Ref
Transmission intensity	Tb Fiber-PZF	Tb Fiber	20 long	0.02–3.2 T	0.49 rad/T	[15]
Beat frequency shift	DBR	Fiber grating	20 long	—	32.5 kHz/mT	[19]
Frequency response	QWP-MO-mirror	MO polymer	15 long	—	20 fT/√Hz	[22]
State of polarization	Silica Fiber	Silica Fiber	—	0–1.5 T	100 mT	[23]
Wavelength shift	Terfenol-D-FBG	Terfenol-D	Φ 4.7 × 20	—	—	[29]
Wavelength shift	Terfenol-D-FBG	Terfenol-D	Φ 5 × 10	0–16.47 mT	4.88 mT	[30]
Wavelength shift	Terfenol-D-PS-FBG	Terfenol-D	20 × 3 × 3	2.43–22.54 mT	0.023 mT	[31]
Wavelength shift	Terfenol-D-FBG	Terfenol-D	60 × 60 × 60	0–350 mT	—	[32]
Wavelength shift	Terfenol-D composite-FBG	Terfenol-D composite	25 × 4 × 4	0–183 mT	3.71 pm/mT	[33]
Wavelength shift	Terfenol-D composite-FBG	Terfenol-D composite	Φ 1.5 × 7	0–750 mT	3.3 pm/mT	[34]
Wavelength shift	Terfenol-D composite-FBG	Terfenol-D composite	50 long	0–300 mT	6 pm/mT	[35]
Beat frequency shift	Terfenol-D composite-HiBi-PCF	Terfenol-D composite	50 × 10 × 10	0–300 mT	10.5 kHz/mT	[36]
Wavelength shift	Terfenol-D film- FBG	Terfenol-D films	Φ 0.085 × 15	0–50 mT	0.9 pm/mT	[38]
Ring-down time spectroscopy	Terfenol-D film-FBG-FPI	Terfenol-D film	Φ 0.125 × 500	0–28.8 mT	0.485 ns/mT	[39]
Wavelength shift	Terfenol-D film-FBG	Terfenol-D film	1.6 um	20–100 mT	0.4 mT	[40]
Wavelength shift	Iron-nickel-FBG	Iron-nickel	Φ 0.185 × 22	0–6 mT	0.75 mT	[41]
Wavelength shift	Terfenol-D-FBG	Terfenol-D	30 long	0–20 mT	0.3 pm/mT	[42]
RBS spectral shift	FeCoV film-fiber	FeCoV films	—	0–143.3 mT	5.3 mT	[45]
Wavelength shift	FBG-MF	MF(Fe_3_O_4_)	—	0–25 mT	3.44 pm/mT	[51]
Transmission spectrum intensity	LFBG-MF	MF(EMG605)	Φ 0.45 × 30	0–11 mT	1.54 dB/mT	[52]
Resonance wavelength shift	LPFG	MF(EMG605)	Φ 1 × 100	0–189.7 mT	176.4 pm/mT	[53]
Resonance wavelength shift	MOF-LFBG-MF	MF(Fe_3_O_4_)	Φ 0.5 × 100	0–72.5 mT	−520 pm/mT	[54]
Extinction ratio	TFBG	MF(EMG605)	—	0–19.6 mT	—	[55]
Transmission spectrum intensity	Etched fiber-MF	MF(Co-ZnO nanorods)	Φ 1 × 420	17–180 mT	—	[56]
Wavelength shift	TMOF-MF	MF (Fe_3_O_4_)	—	0–30 mT	117.9 pm/mT	[58]
Wavelength shift	CCMI-MF	MF (Fe_3_O_4_)	Φ 0.3 × 100	0–21.4 mT	162.06 pm/mT	[57]
Transmission spectrum intensity	TTCF-MF	MF	Φ 1 × 80	4–16 mT	−1.039 dB/mT	[59]
Wavelength shift	MMI-MF	MF (EMG607)	Φ 1 × 30	0–22 mT	−2.93 nm/mT	[60]
Wavelength shift	NATOF-MF	MF (Fe_3_O_4_)	—	0–44 mT	−71.7 pm/mT	[61]
Wavelength shift	NATMF-MF	MF	—	10–22.5 mT	1744 pm/mT	[62]
Transmission spectrum intensity	Up-tapered joints-MF	MF (Fe_3_O_4_)	—	2–30 mT	−0.2121 dB/mT	[63]
Transmission spectrum intensity	IMI-MF	MF (EMG605)	—	0–12 mT	0.106 dB/mT	[65]
Wavelength shift	SNS fiber-MF	MF(EXP08103)	35 long	0–6 mT	6.33 nm/mT	[67]
Wavelength shift	MFC-MF	MF	—	0–240 mT	1718 pm/mT	[69]
Wavelength shift	FPI-FBG-MF	MF (EMG605)	—	20–60 mT	0.53 nm/mT	[70]
Focal line position	Tube filed with MF	MF	—	-	-	[71]
Wavelength shift	SMS fiber-MF	MF (Fe_3_O_4_)	—	12–32.5 mT	−168.6 pm/mT	[72]
Wavelength shift	D-shaped fiber-MF	MF (APGS12n)	—	0.1–30.4 mT	82.3 pm/mT	[73]
Wavelength shift	D-shaped fiber with gold film-MF	MF (EMG 605)	—	0–22.5 mT	5987 pm/mT	[74]
Wavelength shift	PCF-MF	MF	—	0–38.7 mT	110 pm/mT	[75]

**Table A2:** Performance parameters of optical fiber electric field sensors.

Detected Variable	Sensor Probe Configuration	Field Transducer	Probe Size/mm	Detecting Range	Sensitivity/Resolution	Ref
Output light intensity	MZI-LiNbO3	LiNbO3	55 × 2 × 1	0–250 kV/m	2 mV/(kV/m)	[80]
Output light intensity	MZI-LiNbO3	LiNbO3	50 × 10 × 9	0.02–30 kV/m	20 mV/m/√Hz	[83]
Output light intensity	SMF-LiNbO3	LiNbO3	65 × 15 × 15	±801 kV/m	—	[84]
Resonance wavelength shift	Tapered fiber-LiNbO3	LiNbO3	—	<13.16 MV/m	15 kV/m	[85]
Resonance wavelength shift	D-shaped fiber-LiNbO3	LiNbO3	3900 × 27 × 20	<18 MV/m	50 pm/(MV/m)	[85]
Output light intensity	CdTe crystal-Faraday rotator	CdTe	—	—	—	[91]
Output light intensity	ZnTe-Bragg mirror	ZnTe	—	—	—	[92]
Output light intensity	PMPCF-NLC	NLC	30 long	3.4–4.1 MV/m	50 V/m	[94]
Output light intensity	PC cavity-LC	LC	—	2–10 MV/m	0.143 V/m	[97]
Transmission spectrum intensity	MZI-Propylene carbonate	Propylene carbonate	—	5–15 MV/m	∼0.1 W/(V/m)	[98]
Output light intensity	MZI-Propylene carbonate	Propylene carbonate	—	<1400 kV/m	∼89 V/m	[99]
Wavelength shift	FP etalon-NLC	NLC	—	4.494–7.865 kV/m	121 V/m	[100]
Transmission spectrum intensity	TFBG-NLC	NLC	—	100~400 kV/m	2.87 dB/(MV/m)	[104]
Wavelength shift	FBG-PLZT	PLZT	—	0–200 kV/m	-	[106]
Wavelength shift	FBG-PZT	PZT	—	0–400 V/mm	0.45 pm/(kV/m)	[107]
Wavelength shift	FBG-PZT	PZT	—	±30 kV/m	−27 nW/V	[108]
Resonance wavelength shift	FPI-polyester film	polyester film	—	500–1600 kV/m	—	[111]
Membrane movement.	Membrane-photodetector	Coper membrane	—	0–17 kV/m	3 3 μV /(V/m)	[113]
Light flux intensity	MEMS chip	Si plate	6 × 6	<10 kV/m	100 V/m/√Hz	[114]

## Appendix B

This appendix contains the acronyms list used in this manuscript.

Acronym	Definition
AC	Alternating Current
BBO	Barium-Borate
CCMI	Core–Cladding–Mode Interferometer
CdTe	Electro-Optic Crystal
DBR	Distributed Bragg Reflector
DC	Direct Current
DGD	Differential Group Delay
EMF	Electromagnetic Field
FBG	Fiber Bragg Grating
FP	Fabry-Perot
GMM	Giant Magnetostrictive Material
HF	Hydrofluoric
HiBi PCF	Highly Birefringent Photonic Crystal Fiber
IMI	Intermodal Interferometer
LC	Liquid crystal
LFBG	Long-period Fiber Grating
MEMS	Micro Electro Mechanical Systems
MF	Magnetic Fluid
MFC	Microfiber Coupler
MMI	Microfiber Mode Interferometer
MOF	Microstructured Optical Fiber
MZI	Mach-Zehnder Interferometer
NATMF	Nonadiabatic Tapered Microfiber
NATOF	Nonadiabatic Tapered Optical Fiber
NCF	No-Core Fiber
NLC	Nematic Liquid Crystal
OFDR	Optical Frequency Domain Reflectometer
OTDR	Optical Time-Domain Reflectometry
PCF	Photonic Crystal Fiber
PDL	Polarization Dependent Loss
PLZT	Lead lanthanum zirconate titanate
PMF	Polarization Maintaining Fiber
PS-FBG	Phase-Shifted Fiber Bragg Grating
PVDF	Polyvinylidene Fluoride
PZF	Polarization Fiber
PZT	Piezoelectric ceramics
RBS	Rayleigh Backscattering Spectra
SMF	Single-Mode Fiber
SMS	Singlemode–Multimode–Singlemode
SNS	Singlemode-No-core-Singlemode
Tb	Terbium
TFBG	Tilted Fiber Bragg Grating
TMOF	Tapered Microstructured Optical Fiber
TTCF	Tapered thin-core fiber
